# Neighborhood eviction trajectories and odds of moderate and serious psychological distress during pregnancy among African American women

**DOI:** 10.1093/aje/kwae025

**Published:** 2024-03-22

**Authors:** Shawnita Sealy-Jefferson, Benita Jackson, Brittney Francis

**Affiliations:** Division of Epidemiology, College of Public Health, Ohio State University, Columbus, OH 43210, United States; Department of Psychology, Smith College, Northampton, MA 01063, United States; FXB Center for Health and Human Rights, Harvard University, Boston, MA 02115, United States; Harvard Center for Population and Development Studies, Harvard T.H. Chan School of Public Health, Boston, MA 02115, United States

**Keywords:** psychological distress, reproductive justice, residential eviction, neighborhood effects on health, African American women, life-course theory, housing justice, structural racism

## Abstract

African American mothers are unjustly burdened by both residential evictions and psychological distress. We quantified associations between trajectories of neighborhood evictions over time and the odds of moderate and serious psychological distress (MPD and SPD, respectively) during pregnancy among African American women. We linked publicly available data on neighborhood eviction filing and judgment rates to preconception and during-pregnancy addresses from the Life-course Influences on Fetal Environments (LIFE) Study (2009-2011; *n* = 808). Multinomial logistic regression–estimated odds of MPD and SPD during pregnancy that were associated with eviction filing and judgment rate trajectories incorporating preconception and during-pregnancy addresses (each categorized as low, medium, or high, with two 9-category trajectory measures). Psychological distress was measured with the Kessler Psychological Distress Scale (K6) (K6 scores 5-12 = MPD, and K6 scores ≥13 = SPD). MPD was reported in 60% of the sample and SPD in 8%. In adjusted models, higher neighborhood eviction filing and judgment rates, as compared with low/low rates, during the preconception and pregnancy periods were associated with 2- to 4-fold higher odds of both MPD and SPD during pregnancy among African American women. In future studies, researchers should identify mechanisms of these findings to inform timely community-based interventions and effective policy solutions to ensure the basic human right to housing for all.

This article is part of a Special Collection on Mental Health.

Psychological distress is defined as a negative psychological response to stressors which may include nervousness, unhappiness, irritation, and feeling overwhelmed.^1^^,^^2^ Measurement of psychological distress exists on a continuum, including none or low, moderate, and serious mental distress causing significant impairment and meeting the *Diagnostic and Statistical Manual of Mental Disorders* criteria for a mental illness.^3^^,^^4^ Notably, moderate psychological distress (MPD) is an important outcome to study given that it allows for the identification of persons who have clinically relevant (though subdiagnostic) mental distress and could benefit from mental health interventions.^4^ Recent estimates suggest that 3.4% of the overall US adult population has serious psychological distress (SPD), and inequities by gender and race have been documented.^5^ Increases in national rates of suicide, opioid misuse disorder, and associated overdose deaths suggest increasing psychological distress.^6^^-^^8^

African American women are among those who are disproportionately affected by SPD.^5^^,^^9^ Manifestations of structural racism, which is a root cause of racial inequities in population health,^10^^,^^11^ may explain this unequal burden. Defined by scholars as the “totality of ways in which societies foster racial discrimination through mutually reinforcing systems of housing, education, employment, earnings, benefits, credit, media, health care, and criminal justice”,^12^^(p.1453)^ structural racism deserves more empirical attention because documenting the impact on population health is important for identifying solutions.

A key pervasive manifestation of structural racism is racial and economic residential segregation^12^ and concomitant discrimination in the rental and housing markets.^13^ For example, home appraisal discrimination in low-income neighborhoods leads to lower property values, making many of these neighborhoods prime targets for gentrification.^14^^,^^15^ In a vicious cycle, landlords can use various strategies to remove tenants from their units so that they can renovate and charge higher rent. As rent increases in gentrifying areas, tenants may be unable to afford their housing, leading to nonpayment of rent and evictions.^15^ Mass evictions in neighborhoods lead to housing instability, displacement of communities, separation of social networks, and decreased access to resources.^15^^,^^16^ A growing body of literature documents that housing instability caused by residential evictions unjustly impacts African American mothers.^17^^-^^19^

Numerous studies have examined associations between various neighborhood characteristics and mental health outcomes, though studies of the potential spillover effects of neighborhood housing conditions^20^ on individual mental health are few.^21^ A recent review documented the potential pathways through which mental health, including psychological distress, could be negatively affected by neighborhood-level threats of eviction.^22^ Despite possible reverse causation and the dearth of studies evaluating changes over time, a true “neighborhood effect” on mental health is likely,^23^ because living in a neighborhood with high rates of eviction may lead to anticipatory stress about potentially experiencing eviction oneself,^24^ and we would expect personal experiences of forced residential evictions, or the threat of eviction, to have even greater effects.

Structural racism limits the opportunity of African American mothers to have safe, secure, affordable, and sustainable housing, and increases their risk for adverse maternal health outcomes.^25^ Research on macrosocial determinants of health is ever more important given the worsening injustices in African American maternal health.^26^ Researchers have hypothesized that SPD during pregnancy lies on the pathway between disadvantaged neighborhood context and adverse pregnancy outcomes, though this connection has not been conclusively established.^27^ Given the literature on stress, fetal programming, and maternal well-being,^28^^,^^29^ there is a glaring gap in the epidemiologic literature of research leveraging a life-course framework and focused on neighborhood-level eviction as a predictor of MPD and SPD *among* African American women. Many epidemiologic studies document racial inequities in population health with racial group comparisons, with much less attention to within-group empirical analyses, and this has stalled our ability to identify risk and protective factors^30^ among segments of our population that are made vulnerable to poor health and premature mortality by structural racism. Calls have been made for rigorous studies focusing on neighborhood change among different population groups and geographic locations^31^ and for studies that use objective measures of urban neighborhood environments.^32^

Life-course epidemiology requires conceptual rigor and novel data linkages to investigate the accumulation of exposures over time, especially sensitive periods of exposure.^33^ This approach “explicitly require[s] the temporal ordering of exposures and their inter-relationships” in order to appropriately contextualize and statistically quantify relationships of interest.^34^^(p.778)^ Here, we focus on the perinatal and during-pregnancy periods among Black mothers within the context of historical and contemporary structural racism in the United States. A critical and understudied manifestation of structural racism is vicarious racism: instances of prejudice and discrimination that happen to relatives, close friends, or strangers with shared identities.^35^ The “epidemiology of reproductive justice,” a descriptor coined by Professor Loretta Ross, deepens the study of these sensitive periods and further contextualizes vicarious racism. Defined as the interconnected human rights to have children, to not have children, and to parent one’s children in a safe and healthy environment free from individual and/or state violence,^36^ reproductive justice should be the foundation of scholarship on the worsening African American maternal health crisis. Reproductive justice makes clear the importance of understanding the trauma of living in a neighborhood where your neighbors, some of whom may be family members or close friends, experience the threat of eviction or are forced to move because of a court order. Given the gaps in the literature and the potential public health and policy relevance of this issue, our objectives were to examine associations between trajectories of neighborhood eviction filing and judgment rates over time and the odds of MPD and SPD during pregnancy among African American mothers. We hypothesized that both exposure variables, one reflecting the threat of eviction (filings) and the other reflecting the forced displacement of neighbors (judgments), would be detrimental to the mental health of African American mothers.

## Methods

### Sample

This study used data collected from the Life-course Influences on Fetal Environments (LIFE) Study, which has been described elsewhere.^37^ Briefly, the LIFE Study is a retrospective cohort study of African American women aged 18-45 years who were enrolled between 2009 and 2011 at a suburban hospital in metropolitan Detroit, Michigan (*n* = 1410, which represents 71% of the women who were invited to participate). LIFE was originally conducted to determine whether and how racism is associated with preterm birth in African American women. Exclusion criteria included being non–English-speaking, having intellectual disabilities, having serious cognitive deficits, or having evidence of mental illness (on the basis of any prior records or history). In-person interviews were completed during the postpartum hospital stay. The current study was restricted to women who reported their address 2 years before study enrollment and at study enrollment, and whose address was able to be matched to a latitude and longitude using ArcGIS 10.8.2 (*n* = 808). Informed consent was obtained from all LIFE Study participants, and all appropriate IRBs approved the LIFE Study. This secondary data analysis of LIFE Study data, as a part of the Social Epidemiology to Combat Unjust Residential Evictions (SECURE) Study, was determined to be exempt from IRB review by the Office of Responsible Research Practices at Ohio State University.

### Exposure ascertainment

Residential eviction cases are usually heard in county-level civil courts, with eviction cases being resolved by either: (1) an eviction judgment, where the tenant receives a time-sensitive order to move from the residence by a judge; (2) dismissal of the eviction case, with the tenant being allowed to remain in the residence; or (3) a mediated agreement between the tenant and landlord in which the eviction is dismissed if the tenant follows a negotiated payment schedule, with an eviction judgment being rendered if the tenant does not adhere to the agreement.^38^ Our exposures of interest included census block-group–level rates of eviction filings and eviction judgments.

The neighborhood-level eviction filing rate is interpreted as the ratio of all eviction cases filed, counting multiple cases filed against the same address in the same year, to the total number of renter-occupied homes in that block group. The neighborhood-level eviction judgment rate is interpreted as the ratio of the number of renter-occupied households in the block group that received an eviction judgment by the court (addresses were only counted once per year) to the total number of renter-occupied homes in that block group. The eviction rates were calculated using the original, nonrounded values to prevent inflation of the estimates, to correspond to the number of evictions per 100 renter-occupied homes and were validated at the individual and aggregate levels. We used block groups to represent neighborhoods and linked publicly available data (standardized to reflect Census 2010 boundaries) on court-ordered eviction rates from 2007-2011 from the Eviction Lab at Princeton University^38^ to the latitudes and longitudes of preconception and during-pregnancy addresses of LIFE Study participants. Study participants reported their addresses before enrollment (considered the preconception period, 2007-2009) and at enrollment (considered the during-pregnancy period, 2009-2011). We matched neighborhood-level eviction rates to LIFE Study participant addresses at 2 time points, based on the year the address corresponded to (eg, preconception eviction rates for 2007 were linked to LIFE Study participants who enrolled in 2009 and reported their address from 2 years before enrollment).

### Outcome ascertainment

Psychological distress during the past 30 days was measured with the Kessler Psychological Distress Scale (K6).^3^ Study participants recounted how often they felt (1) nervous, (2) hopeless, (3) restless or fidgety, (4) so depressed that nothing could cheer them up, (5) that everything was an effort, and (6) worthless, on a 5-point Likert scale ranging from all of the time to none of the time. Individual K6 items were reverse-coded and summed; higher scores represented higher levels of psychological distress, and the range of scores in our analysis was 0 to 24 (mean = 6.67 (SD, 4.09); median, 6). The internal consistency reliability of the K6 was 0.72.

### Covariates

Because where people live is nonrandom,^39^ we adjusted our analyses for the following predictors of residential selection: age (<35 years or ≥35 years), income (dichotomized at <$35 000/y), and educational attainment (≤12 years or >12 years). We also controlled for duration of time lived in the during-pregnancy neighborhood (dichotomized at ≤2 years) and a block-group–level neighborhood disadvantage index used in prior work.^40^

### Statistical analysis

We used univariable and multivariable statistics to describe the data, including χ^2^ and Wilcoxon rank sum tests to examine differences in categorical and continuous variables. We estimated Spearman correlation coefficients (*r*_S_) among neighborhood eviction measures. The prevalence of missing data on all covariates ranged from 0% to 10%. Given that there was little block-group–level variation in psychological distress (which precluded hierarchical modeling) and there was evidence that the proportional odds assumption was not valid in our data, we used multinomial logistic regression to estimate unadjusted and adjusted odds ratios (ORs) and associated 95% CIs for associations between trajectories of neighborhood eviction filings and judgments over time and the odds of (1) MPD (K6 scores 5-12) and (2) SPD (K6 scores ≥13) during pregnancy, as compared with none (K6 scores ≤4).

To examine continued exposure as well as change in exposure^41^ to neighborhood eviction filings and judgments, we categorized each rate at each time point into tertiles (low, medium, or high). Trajectory measures incorporated preconception and during-pregnancy neighborhood eviction filing and judgment rates, and each had 9 categories representing the following preconception/during-pregnancy neighborhood combinations: (1) low/low, (2) low/medium, (3) low/high, (4) medium/low, (5) medium/medium, (6) medium/high, (7) high/low, (8) high/medium, and (9) high/high. Women who lived in neighborhoods with low/low eviction filing and judgment rates were the referent group. We used ArcGIS 10.8.2 to geocode preconception addresses, and during-pregnancy addresses were previously geocoded and linked to data from Princeton’s Eviction Lab.^19^^,^^38^ We used SAS, version 9.4 for Windows, for the statistical analyses.

## Results

The mean age of the sample was 27 years. Over half of the participants were married to or living with the father of their baby, had an income of less than $35 000/y, and had lived in their during-pregnancy neighborhood for less than 2 years. Nearly 70% of the sample had more than 12 years of education, and the majority enrolled in the study during the year 2011 (Table 1). In terms of the prevalence of psychological distress during pregnancy, 30% of the sample reported no psychological distress, 60% reported MPD, and 8% reported SPD.

**Table 1:** Sociodemographic characteristics of study participants by level of psychological distress and odds of moderate and serious psychological distress during pregnancy (bivariate multinomial regression), LIFE Study, 2009-2011.

	**Participant group, no. (%)** ^**a**^				**Comparison** ^**b**^			
		**Level of psychological distress**			**MPD vs none**		**SPD vs none**	
**Characteristic**	**Total sample** **(*n* = 808; 100%)**	**No distress** **(*n* = 240; 29.7%)**	**Moderate distress** **(*n* = 488; 60.4%)**	**Serious distress** **(*n* = 65; 8.0%)**	**OR**	**95% CI**	**OR**	**95% CI**
Age group, y								
18-19	70 (8.7)	16 (6.7)	44 (9.0)	8 (12.3)	1.23	0.64-2.35	1.67	0.61-4.53
20-24	271 (33.5)	67 (27.9)	170 (34.8)	26 (40.0)	1.14	0.75-1.72	1.29	0.65-2.59
25-29	213 (26.4)	60 (25.0)	134 (27.5)	18 (27.7)	1.00	Referent	1.00	Referent
30-34	146 (18.1)	50 (20.8)	88 (18.0)	6 (9.2)	0.79	0.50-1.25	0.40	0.15-1.08
≥35	108 (13.4)	47 (19.6)	52 (10.7)	7 (10.8)	0.50	0.30-0.82	0.50	0.19-1.29
Marital status								
Single	364 (45.3)	92 (38.8)	223 (45.8)	44 (67.7)	1.00	Referent	1.00	Referent
Married/cohabiting	439 (54.7)	145 (61.2)	264 (54.2)	21 (32.3)	0.75	0.55-1.03	0.30	0.17-0.54
Education, y								
≤12	317 (43.7)	61 (25.4)	158 (32.4)	21 (32.3)	1.41	0.99-1.99	1.40	0.77-2.54
>12	561 (69.4)	179 (74.6)	330 (67.6)	44 (67.7)	1.00	Referent	1.00	Referent
Annual income								
<$35 000	409 (56.3)	106 (48.4)	249 (56.7)	43 (78.2)	1.40	1.01-1.93	3.82	1.91-7.64
≥$35 000	317 (43.7)	113 (51.6)	190 (43.3)	12 (21.8)	1.00	Referent	1.00	Referent
Length of residence in during-pregnancy neighborhood, mo								
<24	466 (58.5)	122 (51.7)	296 (61.4)	36 (56.3)	1.00	Referent	1.00	Referent
≥24	331 (41.5)	114 (48.3)	186 (38.6)	28 (43.8)	0.67	0.49-0.92	0.83	0.48-1.45
Year of study enrollment								
2009	102 (12.6)	26 (10.8)	60 (12.3)	14 (21.5)	1.00	Referent	1.00	Referent
2010	239 (29.6)	54 (22.5)	152 (31.2)	21 (32.3)	1.22	0.70-2.13	0.72	0.32-1.64
2011	467 (57.8)	160 (66.7)	276 (56.6)	30 (46.2)	0.75	0.45-1.23	0.35	0.16-0.74

Abbreviations: LIFE, Life-course Influences on Fetal Environments; MPD, moderate psychological distress; OR, odds ratio; SPD, serious psychological distress.

^a^ Missing data: *n* = 15 (1.86%) participants were missing data for psychological distress, *n* = 82 (10%) were missing data for income, *n* = 5 (0.62%) were missing data for marital/cohabiting status, and *n* = 11 (1.36%) were missing data for duration of residence in the during-pregnancy neighborhood.

^b^ “No psychological distress” was the referent group.

In bivariate models (Table 1), older maternal age at birth (≥35 years) was associated with protection against both MPD (OR = 0.50; 95% CI, 0.30-0.82) and SPD (OR = 0.50; 95% CI, 0.19-1.29) during pregnancy. Being married to or living with the father of the baby was associated with protection against SPD during pregnancy in bivariate models (OR = 0.30; 95% CI, 0.17-0.54). Further, in bivariate models, low income (<$35 000/y) was associated with increased odds of both MPD (OR = 1.40; 95% CI, 1.01-1.93) and SPD (OR = 3.82; 95% CI, 1.91-7.64) during pregnancy. Finally, residing in the during-pregnancy neighborhood for at least 24 months was associated with lower odds of MPD during pregnancy (OR = 0.67; 95% CI, 0.49-0.92), and those who enrolled in the year 2011 had lower odds of SPD during pregnancy (OR = 0.35; 95% CI, 0.16-0.74).

All correlations between eviction filing and judgment rates in the preconception neighborhood and the during-pregnancy neighborhood were significantly different from 0 (*P* <.0001) (Table 2). Correlations between eviction filing and judgment rates in the preconception (*r*_S_ = 0.73) and during-pregnancy (*r*_S_ = 0.70) neighborhoods were strongest. The weakest correlation was between preconception eviction filing rates and during-pregnancy eviction judgment rates (*r*_S_ = 0.20). In the preconception period, our study participants lived in neighborhoods with an average eviction filing rate of 24.85 filings (SD, 16.12) per 100 renter-occupied homes, and in the during-pregnancy period they lived in neighborhoods with an average eviction filing rate of 26.14 (SD, 16.53) per 100 renter-occupied homes. The mean preconception neighborhood eviction judgment rate was 10.23 judgements (SD, 7.37) per 100 renter-occupied homes, and for the during-pregnancy neighborhood it was 8.79 (SD, 6.87) per 100 renter-occupied homes. The range of eviction filing rates widened when comparing preconception neighborhoods (0-116.71 per 100 renter-occupied homes) with during-pregnancy neighborhoods (0-132.00 per 100 renter-occupied homes). A similar widening of the range of eviction judgment rates was observed when comparing preconception neighborhoods (0-48.39 per 100 renter-occupied homes) with during-pregnancy neighborhoods (0-59.00 per 100 renter-occupied homes).

**Table 2:** Spearman correlations (*r*_S_) between eviction filing and judgment rates in the preconception neighborhood and the during-pregnancy neighborhood among African American women (*n* = 808), LIFE Study, 2009-2011.

	**Pregnancy period and rate** ^**a**^			
	**Preconception neighborhood**		**During-pregnancy neighborhood**	
**Variable**	**Eviction judgment rate**	**Eviction filing rate**	**Eviction judgment rate**	**Eviction filing rate**
Pregnancy period and rate				
Preconception neighborhood				
Eviction judgment rate	1			
Eviction filing rate	0.73^b^	1		
During-pregnancy neighborhood				
Eviction judgment rate	0.25^b^	0.22^b^	1	
Eviction filing rate	0.20^b^	0.25^b^	0.70^b^	1
Mean (SD)	10.23 (7.37)	24.85 (16.12)	8.79 (6.87)	26.14 (16.53)
Median	8.72	23.21	7.00	25.00
Range	0-48.39	0-116.71	0-59.00	0-132.00

Abbreviation: LIFE, Life-course Influences on Fetal Environments.

^a^ Rate = number of eviction judgments or filings per 100 renter-occupied homes.

^b^ All correlations were significantly different from 0 (*P* <.0001).

For the eviction judgment rate trajectories, the low/low category had the highest percentage of participants (15.7%), and the low/high trajectory had the lowest (8.0%) (Table 3). For eviction filing rate trajectories, the low/low category had the highest proportion of participants (15.5%), and the high/low group had the lowest (7.1%). For eviction filing rates, the medium/medium trajectory had a range of 17.2-31.0 per 100 renter-occupied homes over the two time periods, and the medium/high trajectory had approximately 20 more filings in the during-pregnancy neighborhood, with a range of 17.2-132.0 per 100 renter-occupied homes across the two time points (Table 4). The range of neighborhood eviction judgment rates for the medium/medium trajectory was 6.0-11.8 per 100 renter-occupied homes from the preconception period to the during-pregnancy period. The medium/high eviction judgment trajectory had on average 7 more eviction judgments when comparing preconception neighborhoods with during-pregnancy neighborhoods, with a range of 6.3-11.8 in the preconception neighborhood and 10.0-59.0 per 100 renter-occupied homes in the during-pregnancy neighborhood. The change in eviction judgment rates for the low/medium trajectory was approximately 3 more eviction judgments in the during-pregnancy neighborhood per 100 renter-occupied homes (range, 0-9).

**Table 3:** Distribution of participants according to trajectory of preconception neighborhood and during-pregnancy neighborhood rates of eviction filings and judgments (*n* = 808), LIFE Study, 2009-2011.

**Preconception neighborhood** **rate/during-pregnancy neighborhood rate**	**Rate** ^**a**^ **trajectory, no. (%)**	
	**Eviction** **filings**	**Eviction** **judgments**
Low/low	125 (15.5)	127 (15.7)
Low/medium	71 (8.8)	77 (9.5)
Low/high	73 (9.0)	65 (8.0)
Medium/low	79 (9.8)	84 (10.4)
Medium/medium	110 (13.6)	96 (11.9)
Medium/high	81 (10.0)	90 (11.1)
High/low	57 (7.1)	74 (9.2)
High/medium	88 (10.9)	71 (8.8)
High/high	124 (15.4)	124 (15.4)

Abbreviation: LIFE, Life-course Influences on Fetal Environments.

^a^ Rate = number of eviction judgments or filings per 100 renter-occupied homes.

**Table 4:** Preconception neighborhood and during-pregnancy neighborhood eviction filing and judgment rate tertile categories and associated descriptive statistics (*n* = 808), LIFE Study, 2009-2011.

	**Pregnancy period and tertile of eviction filing or judgment rates** ^**a**^					
	**Preconception neighborhood**			**During-pregnancy neighborhood**		
**Variable**	**Low (T1)**	**Medium (T2)**	**High (T3)**	**Low (T1)**	**Medium (T2)**	**High (T3)**
Eviction filing rate	(*n* = 269)	(*n* = 270)	(*n* = 269)	(*n* = 261)	(*n* = 269)	(*n* = 278)
Mean (SD)	9.3 (5.6)	23.2 (3.7)	42 (14.0)	9.3 (5.7)	24.5 (4.1)	43.6 (13.2)
Median	9.8	23.2	37.9	10.0	24.0	40.0
Range	0-17.2	17.2-29.3	29.3-116.7	0-17.0	18.0-31.0	32.0-132.0
Eviction judgment rate	(*n* = 269)	(*n* = 270)	(*n* = 269)	(*n* = 285)	(*n* = 244)	(*n* = 279)
Mean (SD)	3.5 (1.9)	8.9 (1.5)	18.3 (6.7)	2.9 (1.7)	7.2 (1.1)	16.2 (6.4)
Median	3.7	8.7	16.2	3.0	7.0	14.0
Range	0-6.3	6.3-11.8	11.9-48.4	0-5.0	6.0-9.0	10.0-59.0

Abbreviations: LIFE, Life-course Influences on Fetal Environments; T, tertile.

^a^ Rate = number of eviction judgments or filings per 100 renter-occupied homes.

Compared with women who lived in neighborhoods with low/low eviction filing rates, we observed a greater than 2-fold increase in odds of MPD during pregnancy for both those who lived in neighborhoods with medium/medium trajectories (aOR = 2.45; 95% CI, 1.29-4.66) and those who lived in neighborhoods with medium/high trajectories (aOR = 2.67; 95% CI, 1.31-5.46) (Table 5). Similar associations were observed for eviction judgment rates as predictors of MPD during pregnancy in the medium/medium (aOR = 2.25; 95% CI, 1.16-4.34) and medium/high (aOR = 2.24; 95% CI, 1.11-4.52) trajectories. Odds of SPD during pregnancy were 3-fold higher for women who lived in the medium/medium trajectory of eviction filing rates (aOR = 3.31; 95% CI, 1.04-10.49) and were 4-fold higher for those in the low/medium trajectory for eviction judgment rates (aOR = 4.03; 95% CI, 1.18-13.74), in comparison with those in the low/low group (Table 6).

**Table 5:** Associations between trajectories of neighborhood eviction filing and judgment rates and odds of moderate psychological distress during pregnancy (multinomial logistic regression) among African American women (*n* = 808), LIFE Study, 2009-2011.

	**Eviction filing rate**			**Eviction judgment rate**		
**Tertile of preconception neighborhood rate** ^**a**^ **/** **tertile of during-pregnancy neighborhood rate**	**No. with MPD**	**Unadjusted** **OR (95% CI)**	**Adjusted** ^**b**^ **OR (95% CI)**	**No. with MPD**	**Unadjusted** **OR (95% CI)**	**Adjusted** ^**b**^ **OR (95% CI)**
Low/low	65	1.00	Referent	71	1.00	Referent
Low/medium	40	1.31 (0.70-2.44)	1.20 (0.61-2.35)	45	1.36 (0.72-2.57)	1.42 (0.72-2.79)
Low/high	45	1.86 (0.97-3.56)	1.75 (0.87-3.54)	40	1.41 (0.72-2.75)	1.45 (0.72-2.94)
Medium/low	48	1.57 (0.85-2.89)	1.51 (0.79-2.90)	57	1.57 (0.85-2.90)	1.91 (0.98-3.72)
Medium/medium	73	2.39 (1.32-4.30)	2.45 (1.29-4.66)	68	1.96 (1.07-3.60)	2.25 (1.16-4.34)
Medium/high	55	2.16 (1.15-4.05)	2.67 (1.31-5.46)	60	1.73 (0.94-3.20)	2.24 (1.11-4.52)
High/low	40	2.62 (1.25-5.49)	2.12 (0.98-4.62)	42	1.27 (0.67-2.41)	1.24 (0.62-2.49)
High/medium	60	2.14 (1.16-3.94)	1.74 (0.89-3.43)	42	1.27 (0.67-2.41)	1.39 (0.69-2.79)
High/high	62	1.11 (0.65-1.88)	1.22 (0.67-2.24)	63	0.85 (0.50-1.45)	1.10 (0.62-1.98)

Abbreviations: LIFE, Life-course Influences on Fetal Environments; MPD, moderate psychological distress; OR, odds ratio.

^a^ Rate = number of eviction judgments or filings per 100 renter-occupied homes.

^b^ Adjusted for age, income, education, length of residence in the during-pregnancy neighborhood, and neighborhood disadvantage index.

**Table 6:** Associations between trajectories of neighborhood eviction filing and judgment rates and odds of serious psychological distress during pregnancy (multinomial logistic regression) among African American women (*n* = 808), LIFE Study, 2009-2011.

	**Eviction filing rate**			**Eviction judgment rate**		
**Tertile of preconception neighborhood rate** ^**a**^ **/** **tertile of during-pregnancy neighborhood rate**	**No. with SPD**	**Unadjusted** **OR (95% CI)**	**Adjusted** ^**b**^ **OR (95% CI)**	**No. with SPD**	**Unadjusted** **OR (95% CI)**	**Adjusted** ^**b**^ **OR (95% CI)**
Low/low	7	1.00	Referent	7	1.00	Referent
Low/medium	11	2.48 (0.75-8.18)	1.90 (0.55-6.58)	11	3.37 (1.14-9.92)	4.03 (1.18-13.74)
Low/high	6	3.58 (1.10-11.67)	1.60 (0.39-6.55)	6	2.14 (0.63-7.26)	2.17 (0.51-9.29)
Medium/low	3	2.13 (0.62-7.28)	1.07 (0.27-4.31)	3	0.84 (0.20-3.55)	1.30 (0.28-6.10)
Medium/medium	5	4.25 (1.42-12.69)	3.31 (1.04-10.49)	5	1.46 (0.42-5.13)	1.82 (0.46-7.22)
Medium/high	6	1.70 (0.43-6.67)	1.26 (0.27-5.84)	6	1.75 (0.53-5.84)	1.36 (0.28-6.54)
High/low	9	2.13 (0.46-9.73)	1.45 (0.31-6.87)	9	2.76 (0.90-8.40)	3.11 (0.87-11.05)
High/medium	6	1.93 (0.53-7.00)	1.15 (0.28-4.70)	6	1.84 (0.55-6.14)	2.65 (0.70-10.10)
High/high	12	2.71 (0.96-7.64)	1.95 (0.62-2.24)	12	1.64 (0.59-4.54)	2.35 (0.72-7.71)

Abbreviations: LIFE, Life-course Influences on Fetal Environments; OR, odds ratio; SPD, serious psychological distress.

^a^ Rate = number of eviction judgments or filings per 100 renter-occupied homes.

^b^ Adjusted for age, income, education, length of residence in the during-pregnancy neighborhood, and neighborhood disadvantage index.

## Discussion

The main finding of this study was that living in a neighborhood with higher eviction filing and judgment rates during the preconception and pregnancy periods was associated with increased odds of MPD and SPD during pregnancy among African American women. Specifically, a greater than 2-fold increase in odds of MPD during pregnancy was associated with stable medium and medium/high eviction filing and judgment rates, across the preconception and pregnancy periods. We also observed a 3-fold increase in odds of SPD for women who lived in a stable medium trajectory and a 4-fold increase in odds for those who lived in a low/medium trajectory for eviction judgment rates.

Importantly, prior evidence that SPD risk among African Americans is associated with their relatives’ homelessness highlights the salience of investigating the larger social context in which this group is embedded.^30^ Observing neighbors being evicted likely increases fear of experiencing a similar fate, especially if one has the same landlord as the neighbors experiencing eviction. In a prior study in Michigan using LIFE data, we found higher eviction rates in the neighborhoods our study participants lived in than in the tri-county area, the entire state of Michigan, and the United States as a whole.^19^ It is also important to emphasize the magnitude of changes in eviction filing and judgment rates from the preconception to pregnancy periods that are associated with increased odds of MPD and SPD during pregnancy among African American women in this study. The average change in eviction filing rates from the preconception period to the during-pregnancy period for the medium-to-high trajectory corresponded to approximately 20 more eviction filings per 100 renter-occupied homes. For eviction judgments, the average change from a medium tertile to a high tertile corresponded to about 7 more eviction judgments per 100 renter-occupied homes, and the change from a low tertile to a medium tertile reflected approximately 3 more eviction judgments per 100 renter-occupied homes.

Not surprisingly, a lower income (<$35 000/y) was associated with increased odds of both MPD and SPD during pregnancy, in bivariate models. We also found that older maternal age at birth was associated with lower odds of MPD and SPD during pregnancy, and that being married to or living with the father of the baby and residing in the during-pregnancy neighborhood for at least 2 years were associated with lower odds of SPD and MPD (respectively) during pregnancy, in bivariate models. African American women who have increased risk of poor mental health because they are made vulnerable to a life-course accumulation of adverse contextual exposures that result from structural racism should be the beneficiaries of future research and interventions focused on scalable protective factors, as well as policy change.

A higher percentage of our analysis subsample (from 2009-2011) reported SPD during pregnancy (8%) as compared with the 4.8% overall prevalence estimate from a nationally representative sample from 2008-2012, though ours is similar to rates among the pregnant and postpartum African American women in that analytical sample (7.5% and 6.7% respectively).^42^ Sixty percent of women in our analysis subsample reported MPD, but few studies have documented the prevalence of this important outcome during pregnancy among African American women. Using data from the National Health Interview Survey, Johnson et al^43^ reported that 14.9% of reproductive-age African American women had MPD and 14% had SPD from 2015 to 2016. In a recent nationally representative study of community-dwelling adults, 16% of the African American participants had SPD and 57% had MPD, and it is surprising that the prevalence of MPD in our cohort was higher than that reported for African Americans during the COVID-19 pandemic.^44^ These results highlight the geographic, life-stage, and temporal differences in mental health within African American communities and should inform future individual, community, and society-level interventions.

Critically, taking a life-course perspective, our study focused on maternal perinatal psychological distress, which is related to pregnancy, parenting, or other stressful life events that occurred during pregnancy.^45^ We focused on sensitive periods of development for both the mother and the infant, as the prenatal and immediate postpartum periods represent a time of increased vulnerability to maternal stress,^46^ as well as threats to the physical and neurobiological development of the infant.^47^^,^^48^ Studies show that African American women of reproductive age have unacceptably low rates of mental health care,^42^^,^^43^^,^^49^ and our results suggest that macrosocial interventions focused on addressing the society-level root causes of poor mental health during pregnancy among African American women are warranted.

### Limitations

We did not have data on length of residence in the preconception neighborhood. Additionally, it is possible that the neighborhood-level eviction filing and judgment rates in both time periods could have involved LIFE Study participants themselves. The eviction data used in this study were limited to court-ordered evictions, which do not account for illegal evictions. These illegal evictions include strong-arm lockouts, threats, landlord neglect, etc, and account for approximately 50% of all evictions^50^; this suggests that our large measures of association (even with some estimate imprecision due to the small number of women with SPD) are underestimates. The SECURE Study, which is currently under way, is documenting the magnitude and severity of court-ordered and illegal evictions and will fill important gaps in our knowledge about this source of housing instability, as well as impacts on African American women, families, and communities. Given our study design, we cannot rule out the possibility of reverse causation, and future longitudinal studies with baseline psychological distress assessments are needed to establish temporality.

### Strengths

To our knowledge, this is the first study to have examined whether trajectories of neighborhood eviction rates—importantly, including the preconception period and during pregnancy—as a manifestation of structural racism are associated with odds of MPD and SPD during pregnancy among African American women. We performed a novel data linkage of surveys including residential history data and publicly available neighborhood-level eviction filing and judgment rates on a sizeable cohort of African American women. In an improvement over studies that use administrative data, our participants reported how long they had lived in their during-pregnancy neighborhood, and we were able to adjust for this and relevant individual-level predictors of residential selection, as well as an objective neighborhood disadvantage index. While we cannot infer causality, our study appears to be the first to document associations between neighborhood eviction filing and judgment rate trajectories—beyond single–time-point data—and the odds of MPD and SPD, and we focused on a priority population from a large metropolitan area. This within–racial-group analysis allowed for the identification of a contextual, policy-relevant, and modifiable risk factor for MPD and SPD during pregnancy among African American women.

### Summary

Our research question was grounded in reproductive justice, and we used a life-course approach to identify trajectories of neighborhood eviction filing and judgment rates over time that are associated with increased odds of MPD and SPD during pregnancy in a group made vulnerable by structural racism. The worsening African American maternal health crisis requires urgent action, including efforts to intervene on macrosocial contextual risk factors for adverse maternal mental health.^51^^,^^52^
